# Old Problem, New Concerns: Hypercortisolemia in the Time of COVID-19

**DOI:** 10.3389/fendo.2021.711612

**Published:** 2021-10-05

**Authors:** Agata Berlińska, Renata Świątkowska-Stodulska, Krzysztof Sworczak

**Affiliations:** Department of Endocrinology and Internal Medicine, Faculty of Medicine, Medical University of Gdańsk, Gdańsk, Poland

**Keywords:** hypercortisolemia, cushing’s syndrome, cushing’s disease, coronavirus disease 2019, COVID-19, severe acute respiratory syndrome coronavirus 2, SARS CoV-2, iatrogenic hypercortisolemia

## Abstract

The ongoing coronavirus disease 2019 (COVID-19) pandemic forced a change in the way we provide medical treatment. Endocrinology in the era of COVID-19 had to transform and reduce its vast potential to the absolute necessities. Medical professionals needed to update their clinical practice to provide their patients as much support and as little harm as possible in these increasingly difficult times. International expert statements were published to offer guidance regarding proper care. It was suggested to simplify the diagnostic scheme of hypercortisolemia and to modify the approach to treatment. Hypercortisolemic patients with COVID-19 and iatrogenic hypercortisolemia due to glucocorticoid use are important clinical scenarios – we aimed to provide a cohesive summary of issues to consider.

## Introduction

The ongoing coronavirus disease 2019 (COVID-19) pandemic forced a change in the way we provide medical treatment. Overburdened healthcare systems struggled to carry out urgent medical procedures, not to mention meticulous, time-consuming diagnostics typical for advanced endocrine services. Endocrinology in the era of COVID-19 had to transform and reduce its vast potential.

The pandemic started in December 2019 in the city of Wuhan localized in Central China. It was first reported to the World Health Organization (WHO) in late December of 2019. The WHO declared the new disease a global public health threat of international concern in late January 2020, and in mid-March 2020 marked it as a pandemic. Clinicians and researchers started seeking for potential salvage drugs. Glucocorticoids – especially dexamethasone – gained worldwide attention due to the overall mortality reduction in oxygen-depending patients in the RECOVERY trial (1).

Axial symptoms of COVID-19 include high fever and respiratory distress. Involvement of multiple organs and systems was observed, including cardiovascular, neurological, psychiatric, and gastrointestinal signs and symptoms (2). In most cases, the clinical course of the disease is nonsevere [up to 84% of cases (3)]. Nevertheless, certain groups, such as the elderly, immunocompromised, and patients with chronic illness, are more at risk of developing the severe form of COVID-19.

Hypercortisolemia was a complex and often confusing problem even in the pre-COVID-19 times. Though fulminant cases of Cushing’s syndrome are generally difficult to overlook due to their distinctive clinical and laboratory features, mild autonomous hypercortisolemia or cyclic Cushing’s syndrome can be more challenging to diagnose (4, 5). Most cases of neoplastic hypercortisolemia are ACTH-dependent and arise from hormonal activity of pituitary tumors overproducing corticotropin (ACTH, adrenocorticotropic hormone). Other backgrounds include primary adrenal tumors, and ectopic ACTH secretion (EAS) seen in, for example, bronchial carcinoid or small cell lung cancer (6). Sometimes patients appear cushingoid and/or exhibit laboratory changes typical for hypercortisolemia without underlying neoplasia – such cases may be explained by non-neoplastic/physiological hypercortisolemia (previously referred to as pseudo-Cushing’s syndrome), factitious disorder, or iatrogenic glucocorticoid excess (4, 7).

Patients suffering from hypercortisolemia tend to develop a wide range of metabolic complications, including impaired glucose metabolism [diabetes in 32% of adult patients, impaired glucose tolerance in additional 30.6% (8)], arterial hypertension [80-95% adults (9, 10)], hypercoagulability, and immunodeficiency (11), all of which overlap with abnormalities previously reported as detrimental in COVID-19. As final outcomes in hypercortisolemic individuals are tied with the level of circulating cortisol, optimal control of hormonal hyperactivity remains a staple of care, and in many patients secondary comorbidities resolve once cortisol excess is eliminated. Typically, surgery is the basic modality of treatment, with additional radiotherapeutic and pharmacological options available. Nonetheless, special circumstances of the COVID-19 pandemic forced alternative, not always simpler, approaches to follow-up and treatment.

## Diagnostic Algorithm of Hypercortisolemia in the COVID-19 Era

Early in the course of the pandemic, experts issued a number of statements updating current clinical approach (12–17). Expert opinion on hypercortisolemia focused mainly on: i. quick and efficient triage of patients into low-risk and high-risk groups, ii. urgent care delivery in moderate and severe cases of hypercortisolemia, iii. avoidance of non-essential diagnostic procedures and hospital visits for high-risk patients, iv. deferral of unnecessary diagnostics in milder cases *modo* watch and wait, v. alternative approach to establishing diagnosis and following with treatment, vi. optimal control over comorbidities, vii. prioritization of medical treatment over surgery whenever necessary, viii. development of well-functioning telemedicine and consultation networks, ix. extensive patient education (12).

Patients with Cushing’s syndrome, especially the ones with fulminant and uncontrolled disease, should be considered chronically immunocompromised and metabolically unstable, and therefore require swift care (17). Such individuals have a significant risk of contracting COVID-19 and a range of secondary comorbidities puts them in a high risk group if they become infected (17, 18). It is crucial for them to adhere to the rules and regulations regarding personal safety, self-isolation, and rigorously hygienic lifestyle (12). High-risk patients should avoid unnecessary hospital/clinic visits, and their medical providers should accommodate them with convenient teleconsultations whenever necessary (19). Blood sampling in a medical facility should be limited to a rational minimum. Basic clinical parameters that are easy to check at home, such as blood pressure or capillary glucose, together with clinical evaluation, could help with day-to-day monitoring of the disease. If cases are ubiquitous and/or mild, their in-depth investigation could be reasonably postponed by 3-6 months (12).

**Figure 1:**
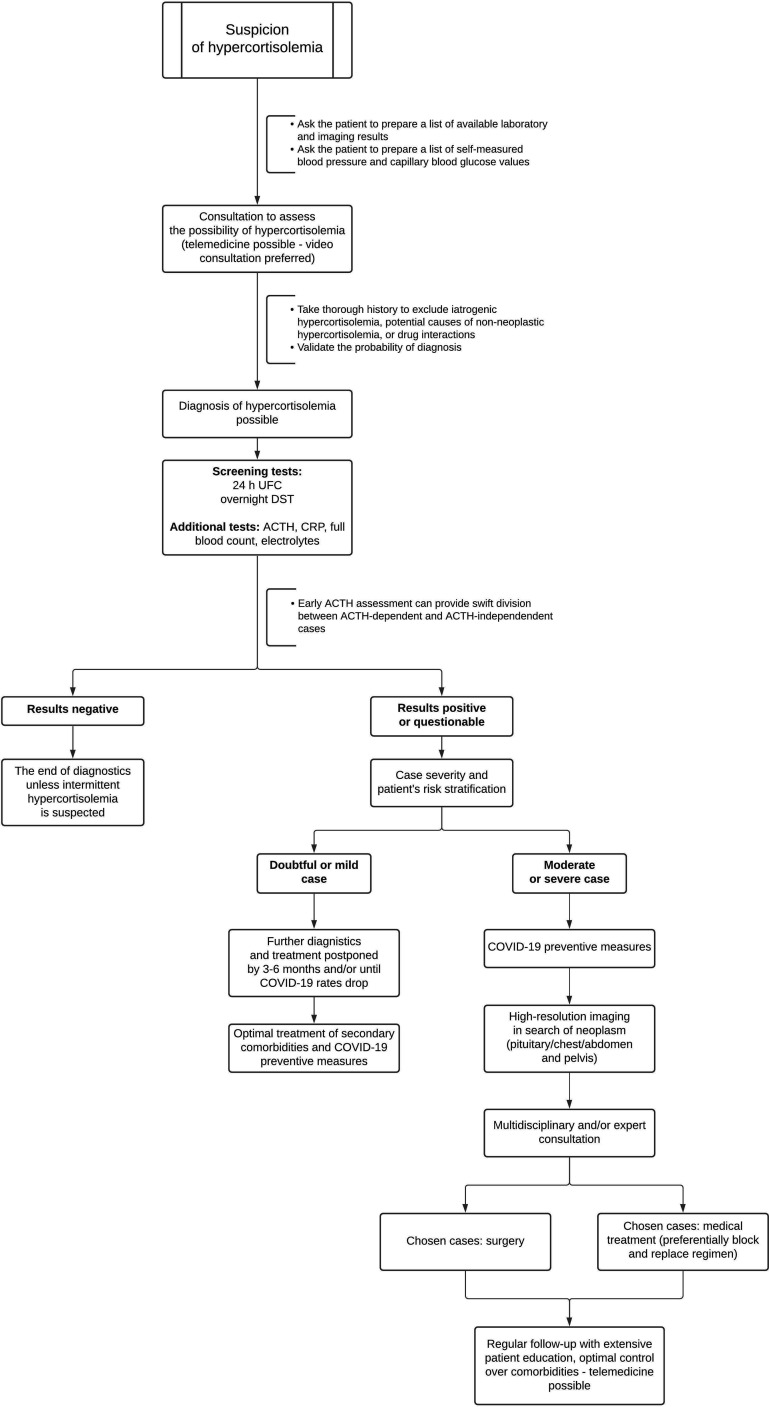
Modified approach to assessment of newly suspected hypercortisolemia in the era of COVID-19 based on the expert guidelines. UFC, urinary free cortisol; DST, dexamethasone suppression test; ACTH, adrenocorticotropic hormone; corticotropin; CRP, C-reactive protein.

Updated diagnostic and therapeutic approach was discussed in detail by Newell-Price et al. and general trends of proposed changes are displayed in **Figure 1** (12). Early assessment of plasma ACTH can help to determine the source of hypercortisolemia (ACTH-dependent *vs*. ACTH-independent). Experts state that in severely symptomatic hypercortisolemia even a single measurement of serum cortisol concentration exceeding 1000 nmol/l (37 µg/dl) could confirm the diagnosis, if only pathologies provoking severe physiological stress are excluded. Similarly, diagnosis of hypercortisolemia is highly likely if urinary free cortisol (UFC) exceeds the upper limit of the norm at least five times. Though the usual first line of tests includes late-night salivary cortisol, during the pandemic this step should be omitted due to possible contamination of the material with SARS CoV-2 copies (12).

## Medical Treatment of Hypercortisolemia and Secondary Comorbidities

Steady drop in circulating cortisol levels together with optimal control over secondary comorbidities should be the primary goals of medical treatment. Physicians can choose between steroidogenesis inhibitors, pituitary- and/or ectopic-source- directed agents, glucocorticoid receptor antagonists, and chemotherapeutics. Medications should be chosen based on their onset of action, safety profile, local availability, monitoring possibility, and the cause of hypercortisolemia. Block-and-replace therapy with steroidogenesis inhibitors and replacement glucocorticoids (and mineralocorticoids whenever necessary) might be introduced *de novo* in steroidogenesis-inhibitor naïve patients, and previously established efficient pharmacological regimens should not be routinely changed, unless it is a switch to block-and-replace therapy to prevent hypoadrenalism (12). Possible interactions between drugs used in Cushing’s syndrome and approved in COVID-19 by the WHO are listed in **Table 1** (21–25). We might be lacking extensive information about potential side effects of some approved drugs such as casirivimab/imdevimab or sotrovimab – due to their novelty (26, 27). Medications showing no clear benefit from treatment – like chloroquine or hydroxychloroquine – were recommended against (21, 28).

**Table 1:** An overview of medical treatment commonly used in neoplastic hypercortisolemia.

Drug group	Drug name	Clinical indication	Clinical concerns	Possible adverse effect accumulation and interactions with drugs used for COVID-19 treatment
Steroidogenesis inhibitors	Metyrapone	Neoplastic hypercortisolemia – all forms	•**Quick onset of action** •Hypertension, hypokalemia •Hirsutism, sexual dysfunction •Liver dysfunction •Therapy monitoring may be difficult due to accumulation of cross-reacting precursors – use tandem mass spectrometry or non-cross-reactive cortisol assay •Hypoadrenalism	•**Remdesivir:** liver dysfunction •**Dexamethasone:** hypertension, hypokalemia •**Baricitinib with dexamethasone:** liver dysfunction, hypertension, hypokalemia •**Tocilizumab with dexamethasone:** liver dysfunction, GI distress, hypertension, hypokalemia
	Ketoconazole	Neoplastic hypercortisolemia – all forms	•**Quick onset of action** •Liver enzyme monitoring essential – potential liver dysfunction •Drug-to-drug interactions – potent inhibitor of CYP3A4 •QT interval prolongation •Optimal absorption form the GI tract requires acidic environment	•**Remdesivir:** liver dysfunction •**Dexamethasone:** increase of plasma concentration of glucocorticoids due to CYP3A4 interaction •**Baricitinib with dexamethasone:** liver dysfunction
	Mitotane	Neoplastic hypercortisolemia – all forms, most commonly used in adrenocortical carcinoma	•Adrenolytic agent, stored and released by adipose tissue •Slow onset of action, requires dose titration •GI distress, particularly nausea and vomiting, and liver dysfunction •Hypoadrenalism •Teratogenic	•**Remdesivir:** GI distress •**Baricitinib with dexamethasone:** liver dysfunction, GI distress •**Tocilizumab with dexamethasone:** liver dysfunction, GI distress
	Osilodrostat	Neoplastic hypercortisolemia – all forms, especially useful in patients with persistent disease	•QT interval prolongation •GI distress, particularly nausea •Recurring headache •Hypoadrenalism	•**Remdesivir:** GI distress •**Baricitinib with dexamethasone:** GI distress •**Tocilizumab with dexamethasone:** liver dysfunction, GI distress, headache
	Etomidate	Neoplastic hypercortisolemia – all forms	•**Quick onset of action** •Intravenous drug, close monitoring required, preferentially within ICU •Hypoadrenalism •GI distress •Myoclonus	•**Remdesivir:** GI distress •**Baricitinib with dexamethasone:** liver dysfunction, GI distress •**Tocilizumab with dexamethasone:** liver dysfunction, GI distress
Pituitary-directed agents	Cabergoline	Neoplastic hypercortisolemia – pituitary tumors	•GI distress, particularly nausea, liver dysfunction •Recurring headache •Orthostatic hypotension •Stops lactation •Long-term use can lead to tissue fibrosis – heart valves included •Long onset of action, should not be used as monotherapy in severe hypercortisolemia	•**Remdesivir:** liver dysfunction, GI distress, headache •**Baricitinib with dexamethasone:** liver dysfunction, GI distress •**Tocilizumab with dexamethasone:** liver dysfunction, GI distress
	Pasireotide	Neoplastic hypercortisolemia – pituitary tumors, ectopic ACTH secretion (if somatostatin receptors are present)	•Hyperglycemia •GI distress, particularly diarrhea, liver dysfunction •QT interval prolongation •Hypoadrenalism •Long onset of action, should not be used as monotherapy in severe hypercortisolemia	•**Remdesivir:** liver dysfunction, GI distress •**Dexamethasone:** hyperglycemia •**Baricitinib with dexamethasone:** liver dysfunction, GI distress •**Tocilizumab with dexamethasone:** liver dysfunction, GI distress
Glucocorticoid receptor antagonists	Mifepristone	Neoplastic hypercortisolemia – all forms	•Symptom-focused peripheral action – does not resolve the origin of hypercortisolemia •Hypertension, hypokalemia •Pharmacological abortion	•**Dexamethasone:** hypertension, hypokalemia •**Baricitinib with dexamethasone:** hypertension, hypokalemia (dexamethasone) •**Tocilizumab with dexamethasone:** hypertension, hypokalemia (dexamethasone)
Chemotherapeutics	Mitotane	As described above	•As described above	•As described above
	Temozolomide	Neoplastic hypercortisolemia – especially in aggressive pituitary tumors	•GI distress, liver dysfunction •Bone marrow suppression •Teratogenic	•**Remdesivir:** liver dysfunction, GI distress •**Baricitinib with dexamethasone:** liver dysfunction, GI distress, neutropenia •**Tocilizumab with dexamethasone:** liver dysfunction, GI distress, thrombocytopenia

GI, gastrointestinal; ICU, intensive care unit; ACTH, adrenocorticotropic hormone; CYP3A4, cytochrome P 3A4.

Partially based on (20).

Hypertension secondary to hypercortisolemia is common (up to 80-95% of hypercortisolemic adults) and multifactorial (9, 10). Its severity is tied to the length of exposure and degree of cortisol excess (29). Mineralocorticoid-like action of glucocorticoids, vascular remodeling, local disruption of synthesis of vasoactive agents, insulin resistance, sleep apnea, and catecholamine hypersensitivity play a detrimental role in development of hypertension (10, 29, 30). In many cases hypertension fades once hormonal stabilization is reached (30). Lifestyle changes, such as weight loss, smoking cessation, and physical activity, remain a staple in non-pharmacological treatment. Due to disturbed renin-angiotensin-aldosterone (RAA) system and progressive vascular and cardiac remodeling, angiotensin-converting enzyme inhibitors (ACE-Is) and angiotensin receptor blockers (ARBs) are perceived the drugs of choice by many physicians (29, 31). Virtually any antihypertensive medication could find its place in the treatment of hypercortisolemia-induced hypertension, however some may be preferred over others due to their cardioprotective properties or potency to wash out or spare serum potassium (10, 29, 31). At the beginning of the pandemic, certain controversies arose around ACE-Is and ARBs since ACE2 receptors were identified as entrance points for the virus. Nonetheless, currently available data proves a high profile of safety of these groups in COVID-19 and advises their continuous use (21, 32–35). Metyrapone can further exacerbate hypertension (**Table 1**) (29).

Up to 60% of hypercortisolemic patients develop glucose metabolism impairment (8). Postprandial hyperglycemia is especially common and blood glucose levels tend to peak in the afternoon or early evening (36). Multiple mechanisms are involved, with hepatic and skeletal muscle glucose homeostasis disruption, disturbed insulin secretion, and insulin resistance (37, 38). Optimal glycemic control can be achieved after resolution of hypercortisolemia. General pharmacological management does not necessarily differ from that proposed for non-hypercortisolemic patients (38, 39). A whole variety of drugs, including metformin, sulfonylureas, acarbose, dipeptidyl peptidase-4 (DPP4) inhibitors, insulin, and others, can be used (38). A potential of dipeptidyl peptidase 4 (DPP4) inhibitors to negatively affect clinical course of COVID-19 was disputed at the beginning of the pandemic. However, prolonged observation showed that DPP4 inhibitor use reduces COVID-19 mortality among diabetic patients (40, 41) and that these drugs should not be routinely withdrawn (42–44). Pasireotide may further provoke hyperglycemia (**Table 1**) (45).

Additional steps in medical treatment include antithrombotic prophylaxis (17, 46, 47) and antibiotic prophylaxis with trimethoprim+sulfamethoxazole to prevent opportunistic *Pneumocystis jiroveci* infection in severe cases of hypercortisolemia (12, 18, 48).

## Surgical Treatment of Hypercortisolemia

Typical approach to corticotropinomas revolves around surgical excision by an experienced pituitary surgeon (49, 50). Nevertheless, current concerns highlight the risk of transsphenoidal surgery amidst the pandemic for both surgical teams and patients (12, 17, 51). Aerosol formation throughout the procedure could potentially lead to further spread of the disease (12, 17). As most pituitary masses are rather stagnant and display a relatively small growth potential, it was suggested that patients could receive medical treatment as a bridge therapy while awaiting surgery (**Table 1**) (12, 17). Nonetheless, certain scenarios, such as risk of vision loss, highly aggressive tumors creating a significant mass effect, or fulminant hypercortisolemia responding poorly to medical treatment, should trigger appropriate actions, surgery included (12, 17). In cases of rare aggressive macroadenomas, alternative approaches minimizing the risk of droplet formation, like supraorbital craniotomy, can be considered (12, 52). Patients should be screened for SARS CoV-2 infection before the surgery, and their anti-COVID-19 vaccination status should be checked. Besides the aforementioned pituitary tumors, adrenocortical carcinoma and EAS call for urgent surgical care due to their malignant behavior and often fast progression.

## Radiotherapy in Hypercortisolemia

If the tumor growth and/or hormonal activity can be no longer managed by debulking surgeries or medical therapies, or when residual mass shows features typical for an aggressive neoplasm, pituitary radiotherapy can be introduced. Stereotactic radiosurgery (SRS) or fractioned external beam radiotherapy (EBRT) can be optimal options (53). However, radiotherapy sessions require subsequent hospital visits and may put the patient at risk of in-hospital COVID-19 transmission.

## Telemedicine

Telemedicine became essential and effective in providing medical care, especially in at-risk population, those with active COVID-19, and/or those attending follow-up visits (19). Videoconferences can facilitate visual evaluation. Patients should be taught proper techniques of blood pressure and capillary blood glucose measurement. If steroidogenesis inhibitors are started, individuals should be informed about the possibility of developing hypocortisolism and learn about its clinical picture. Education regarding stress-dosing of glucocorticoids (preferably also in written form) should be offered; patients should be equipped with a dose of parenteral glucocorticoids and instructed how to apply it. If the block-and-replace tactic is chosen, its background should be discussed in detail.

## Special Circumstance: Hypercortisolemic Patient With COVID-19

To our best knowledge, cases of patients suffering from endogenous hypercortisolemia and COVID-19 were rarely reported (20, 54, 55), therefore many considerations may be only hypothesized. Hypercortisolemia induces persistent low-grade inflammation and immunosuppression. Immune dysfunction in Cushing’s syndrome originates from defective immune reaction and regulation, as well as immune cell apoptosis (18). Natural immune barriers, such as for example skin, can be disrupted. Hypercortisolemia needs to be treated urgently whenever a life-threatening infection ensues (56). Viral infections in the course of Cushing’s syndrome are often severe and prolonged (18, 57). Initial signs and symptoms of COVID-19 in this group of patients can be misleading, and the typical combination of fever and dyspnea may be absent (17, 54). Therefore, other features like diarrhea, anosmia, dysgeusia, and cough should be assessed (17). If antiviral drugs are started, it is suggested that immunocompromised patients may require prolonged therapy (17, 57–60). Hypertension and diabetes, common sequelae of hypercortisolemia, are known negative prognostic factors in COVID-19, therefore optimal treatment should be introduced early (17). Secondary infections may not generate typical signs and symptoms as well. Infected individuals should be carefully and methodically assessed for superimposed secondary infections and treated accordingly. Opportunistic pathogens are not uncommon in Cushing’s syndrome (18, 57). Whenever a superimposed infection is suspected, routine laboratory essays such as CRP, lactate, procalcitonin, leukocyte count, urinalysis, blood/urine/sputum samples should be obtained, with an optional chest X-ray or CT if indicated. Frail skin should be inspected in search of wounds – possible gates for infection (57). The degree of hypercortisolism can predict the final outcomes, with severe hypercortisolism putting patients at risk the most (61).

Immunocompromise can be reversed only once hypercortisolemia is sufficiently treated, and if the disease-specific drugs are continued, physicians should pay special attention to possible drug-to-drug interactions, risk of organ damage, and side effects(**Table 1**) (20). Effective therapeutic schemes should not be routinely changed (12) unless serious concerns ensue. Routine screening of disease activity in the course of an acute illness is challenging: corticosteroids are physiological stress hormones and their levels rise as a part of the “fight or flight” response. In the acute phase of infection, we suggest using simple indicators of disease control, such as clinical state of the patient, blood pressure, heart rate, serum/capillary glucose, serum electrolytes, and total blood count, instead of more elaborate and perhaps misleading targets. Patients using steroidogenesis inhibitors can eventually develop hypoadrenalism and clinical suspicion of such scenario was already reported in a patient with Cushing’s disease and COVID-19 from Italy (20). Simple parameters such as blood pressure, heart rate, capillary glucose, and serum electrolytes can be used as predictors of possible hypoadrenalism. If suspicion of hypoadrenalism seems valid, stress dosing of glucocorticoids should be introduced, with intravenous infusion readily available at site (12). If the patient’s condition seems stable and ambulatory treatment remains the preferred option, “sick day rules” should be strictly followed. If a particular need for hormonal assessment exists, in our opinion UFC might be helpful as it provides integrated information about cortisol excess over a period of 24 hours contrary to serum cortisol measurement covering a single point in time. Metyrapone or exogenous steroids can alter the results of hormonal tests owing to assay interactions (**Table 1**).

Hypercortisolemia, acute inflammatory disorders, and immobility are all well recognized factors promoting clot formation. Therefore, antithrombotic prophylaxis should be introduced (17, 47), and deep vein thrombosis and/or pulmonary embolism should be adequately treated if only they occur.

As Cushing’s syndrome leads to often dramatic catabolism, sarcopenia, truncal obesity, and osteoporosis, it seems probable that the patients might experience respiratory fatigue and require higher doses of oxygen and/or ventilation support earlier on than their non-hypercortisolemic counterparts.

Vitamin D became known as a natural immunomodulatory compound (62); hence, its levels should be promptly assessed in COVID-19 patients and supplementation should be started if necessary. Vitamin D level was linked to clinical outcomes in COVID-19 (63). General nutrition status which is commonly hampered in hypercortisolemic subjects can be tied to final outcomes as well – the higher the nutrition risk, the worse the prognosis in COVID-19 (64). This, together with concerns over balanced diabetic diet whenever indicated, require a consultation from an experienced dietician. Sarcopenia and osteoporosis might worsen due to transient immobility, decreased physical activity, and possibly detrimental effect of proinflammatory cytokines (58–60) and require urgent rehabilitation. Post-COVID-19 recovery of hypercortisolemic patients may be long and challenging; some patients may experience the debilitating post-acute COVID-19 syndrome (57).

COVID-19 survivors often experience various kinds of psychological and/or psychiatric trauma (65), posttraumatic stress disorder included (66), and hypercortisolemic patients are especially prone to mental distress (67); a consultation with an experienced mental health specialist might be beneficial.

To sum it up, we suggest i. close monitoring of possible signs and symptoms of COVID-19, ii. continuation of cortisol-lowering therapy if only possible, iii. optimal control over secondary comorbidities, iv. prevention of thromboembolic events, v. early assessment of superimposed infections, often opportunistic, vi. nutritional assessment and early dietary intervention, with special regard for vitamin D, vii. early detection of signs and symptoms of hypoadrenalism, viii. support of a multidisciplinary team.

## Special Circumstance: Iatrogenic Hypercortisolemia

The RECOVERY trial proved efficacy of synthetic steroid dexamethasone in improving the outcomes in COVID-19 patients requiring oxygen therapy or mechanical ventilation (1). The proposed regimen consisted of 6 mg of oral or intravenous dexamethasone once daily for up to 10 days (1). Other steroids, such as hydrocortisone or methylprednisolone, were investigated with similar results (68, 69). Physicians must vary prolonged treatment with glucocorticoids, especially in high doses, to prevent the development of iatrogenic hypercortisolemia. Whenever glucocorticoids are used, the approach should focus on using the lowest effective doses for the shortest time possible.

Iatrogenic hypercortisolemia can take the same toll on the body as endogenous steroid excess, with identical complications including hypertension, glucose metabolism impairment, clot formation, infections, sarcopenia, osteoporosis, and mental distress. Secondary comorbidities should start to fade once steroids are discontinued, but before that happens each disease should be properly treated. If the risk of glucocorticoid-induced osteoporosis is high, vitamin D supplementation should be started (70). Proper physical activity/rehabilitation and nutrition can help to resolve sarcopenia (71, 72).

Glucocorticoid cessation requires special attention, especially if the drugs were used for a long time, due to the risk of adrenal gland atrophy. Slow reduction of glucocorticoid doses and early detection of features typical for adrenal insufficiency are essential. The post-acute COVID-19 syndrome shares some characteristics with hypoadrenalism [for example fatigue, tachycardia, and mood changes (73, 74)] and physicians must make sure to distinguish between the two. Simple biochemical investigation including serum electrolytes, total blood count, glucose, cortisol, and plasma ACTH could aid the diagnosis. Adrenal insufficiency can be lethal, therefore proper investigation should never be omitted.

Our suggestions for this subgroup of patients include: i. glucocorticoids used in the lowest effective doses for the shortest time possible; ii. evaluation for signs and symptoms of hypoadrenalism after discontinuation of glucocorticoids; iii. optimal control over secondary comorbidities; iv. prevention of thromboembolic events; v. early assessment for superimposed infections; vi. optimal rehabilitation and nutrition, with special regard for vitamin D status.

## Summary

COVID-19 destabilized global healthcare and forced medical professionals to provide treatment in previously uncommon manners. Multiple expert opinions were published early in the course of the pandemic to help with proper care. The proposed diagnostic algorithm of hypercortisolemia was simplified. It became essential to identify cases requiring urgent medical attention and to offer watchful waiting to mild or doubtful cases. Medical treatment of hypercortisolemia and secondary comorbidities became especially important. Hypercortisolemic patients with COVID-19 and the possibility of iatrogenic hypercortisolemia due to prolonged glucocorticoid use should be given special attention. Hopefully, in the wake of new treatment options for COVID-19 and widespread vaccination programs, the pandemic will finally come to an end.

## Author Contributions

AB – concept of the manuscript, literature review, drafting of the manuscript. RŚ-S – literature review, drafting of the manuscript, critical review of the manuscript. KS – critical review of the manuscript. All authors contributed to the article and approved the submitted version.

## Conflict of Interest

The authors declare that the research was conducted in the absence of any commercial or financial relationships that could be construed as a potential conflict of interest.

## Publisher’s Note

All claims expressed in this article are solely those of the authors and do not necessarily represent those of their affiliated organizations, or those of the publisher, the editors and the reviewers. Any product that may be evaluated in this article, or claim that may be made by its manufacturer, is not guaranteed or endorsed by the publisher.
